# Invaded Invaders: Infection of Invasive Brown Treesnakes on Guam by an Exotic Larval Cestode with a Life Cycle Comprised of Non-Native Hosts

**DOI:** 10.1371/journal.pone.0143718

**Published:** 2015-12-23

**Authors:** Elden T. Holldorf, Shane R. Siers, Jonathan Q. Richmond, Page E. Klug, Robert N. Reed

**Affiliations:** 1 Cherokee Services Group LLC, contracted to U.S. Geological Survey, Yigo, Guam, United States of America; 2 Department of Fish, Wildlife, and Conservation Biology, Colorado State University, Fort Collins, Colorado, United States of America; 3 Western Ecological Research Center, U.S. Geological Survey, San Diego, California, United States of America; 4 Fort Collins Science Center, U.S. Geological Survey, Fort Collins, Colorado, United States of America; Institute of Tropical Medicine, JAPAN

## Abstract

**Background:**

Multiple host introductions to the same non-native environment have the potential to complete life cycles of parasites incidentally transported with them. Our goal was to identify a recently detected parasitic flatworm in the invasive Brown Treesnake (*Boiga irregularis*) on the remote Pacific island of Guam. We considered possible factors influencing parasite transmission, and tested for correlations between infection status and potential indicators of host fitness. We used genetic data from the parasite and information about the native ranges of other possible non-native hosts to hypothesize how it arrived on Guam and how its life cycle may be currently supported.

**Methods:**

We identified the parasite by comparing larval morphology and mtDNA sequences with other Pseudophyllid tapeworms. We assessed probability of infection in individual snakes using logistic regression and examined different factors influencing presence of parasites in hosts.

**Results:**

We identified the parasite as the pseudophyllid cestode *Spirometra erinaceieuropaei*, with all sampled worms from multiple snakes sharing a single mtDNA haplotype. Infection appears to be limited to the only freshwater watershed on the island, where infection prevalence was high (77.5%). Larger snakes had a higher probability of being infected, consistent with the chronic nature of such infections. While infection status was positively correlated with body condition, infected snakes tended to have lower intra-peritoneal fat body mass, potentially indicating a negative effect on energy stores.

**Conclusions:**

We discovered that *B*. *irregularis* inhabiting a small area of forested habitat in a freshwater watershed on Guam are often infected by a novel parasite of Asian origin. While further work is needed, this species of *Spirometra*, itself a non-native species, likely depends on a suite of recently introduced hosts from different parts of the world to complete the life cycle. This baseline study provides little evidence of any effects on host fitness, but additional data are needed to more thoroughly explore the consequences of infection in this invasive snake population.

## Introduction

Non-native species that have invaded areas outside their historic range often escape their co-evolved parasites [1–2]. While there are many ways that introduced hosts might escape parasites, two common, non-mutually exclusive pathways for parasite escape may be that (1) a small number of founder individuals harbored few or none of the parasites from the native range during their initial transportation, or (2) the various hosts and environments required for a parasite to complete its life cycle may not be available in the invader’s new range. In addition to this escape from parasites, the time to which an invasive host acquires new parasites will vary depending on a variety of factors. Hosts experiencing longer bouts of a parasite-free existence may ultimately have enhanced ecological and reproductive success in the introduced range [2–3].

Unlike the native range where parasite and host diversity are closely linked by their co-evolutionary history, a host that invades a new area may experience a shift in this association such that it becomes a new resource for different parasites in the introduced range [4]. Hosts that invade new areas also have the potential to alter trophic links within food webs, particularly if they harbor parasites whose life cycles can be supported in the introduced range [5].

Few studies have documented parasite infection in Brown Treesnakes (*Boiga irregularis*), an iconic invasive species due to its ecological and economic impacts on the island of Guam [6–7]. *Boiga irregularis* is native to Indonesia, coastal Australia, and islands throughout northwest Melanesia, and was presumably introduced to Guam via military cargo during the 1940s [8]. Recent research has confirmed a longstanding hypothesis that the invasive population of *B*. *irregularis* originated from Manus Island in the Admiralty Islands archipelago of Papua New Guinea and was likely established by fewer than 10 individuals [9].

Although the most notorious invasive species on Guam is the *B*. *irregularis*, the island’s terrestrial community is currently dominated by a diversity of introduced species, from flora to invertebrates to mammals, and has been cited as an example of invasional meltdown [10–13]. Guam provides a clear example of the consequences of increasing global transportation of goods and people, as it is an air and sea transportation hub in the Pacific. On occasion, this movement has resulted in the concurrent transportation of plants and animals from outside areas, and over time has produced a novel assemblage of invasive species on the island. This in turn has provided novel habitat for introduced parasites to exploit.

Parasites have important implications for invasion biology as they may regulate population densities, negatively affect host fitness, mediate competition, and alter food webs [3, 14–16]. A survey of blood, fecal and ectoparasites from 33 native, terrestrial snake species in Australia and the Solomon Islands (including *B*. *irregularis*) recovered at least one species of parasite in 100% of Colubrid species (N = 5), 100% of Boidae species (N = 10), and 50% of Elapidae species (N = 18), with prevalence in wet tropical regions exceeding 80% of individuals sampled [17]. In this study, native *B*. *irregularis* (N = 87) harbored at least two species of ectoparasites, three or more species of haemogregarine-like blood protozoa, two types of fecal protozoa, and numerous nematode and cestode species. No parasites were found in samples of invasive *B*. *irregularis* on Guam (N = 22), however researchers did not collect fecal samples from the invasive population. Richmond *et al*. [18] compared parasite abundance and diversity in native populations of *B*. *irregularis* (Papua New Guinea and the Admiralty Islands) to the invasive population on Guam and reported fewer infected snakes and notably lower infection intensities on Guam (24% of individuals positive for helminth infections on Guam compared to 88% in the native range), conditions consistent with the hypothesis of enemy release. Both studies found no evidence of haemogregarine infections in *B*. *irregularis* on Guam, compared with native infection prevalence as high as 75% in some areas of Australia. With a reduced rate and intensity of parasitism, *B*. *irregularis* on Guam have likely enjoyed fitness benefits; however, circa 60 years after its introduction to Guam, this alien population may now be accumulating novel parasites.

Starting in 2010, we began to observe a species of cestode (Class: Cestoda) in *B*. *irregularis* collected from a geographically isolated area on Guam that contains freshwater habitat. In this study we offer the first report on the species identity of this cestode and present data on its prevalence and distribution based on an island-wide sample of snakes. We also conducted a more rigorous screening of a subsample of snakes collected from the known area of infection on Guam. For this subsample, we investigated individual and environmental factors influencing whether or not snakes were infected and made preliminary assessments of the association between infection and body condition or reproductive status. We conclude by discussing the geographic source of the parasite and how the various stages of its life cycle could be currently supported on Guam.

## Methods

### Brown Treesnake Sampling and Screening

**Fig 1:**
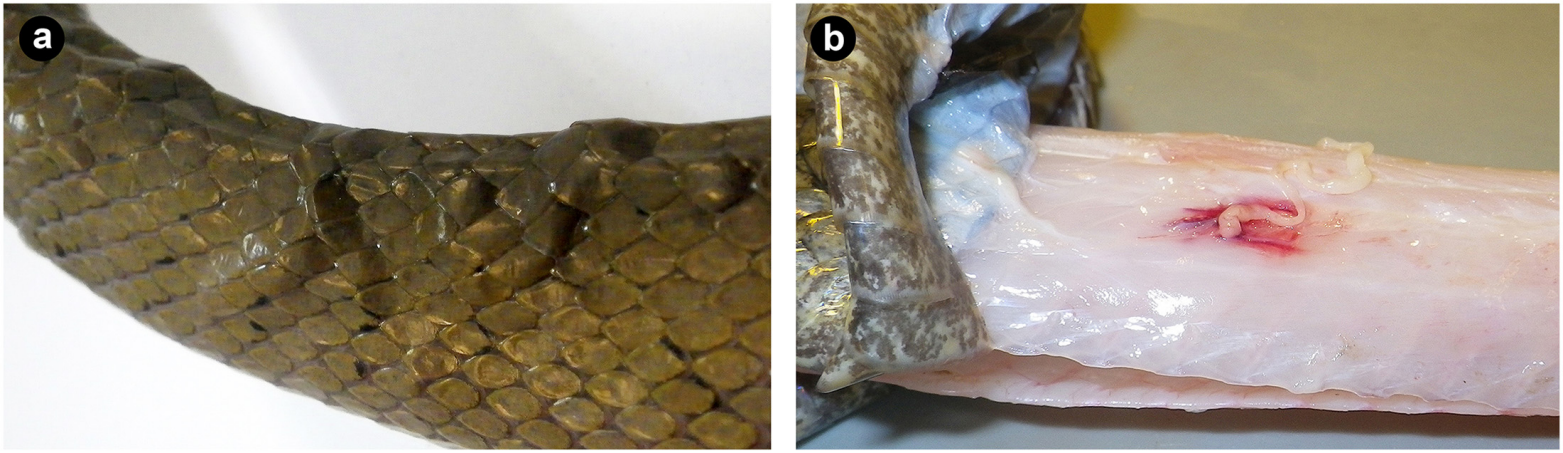
Pathological signs of *Spirometra erinaceieuropaei* infection in *Boiga irregularis*. (a) External blister-like nodules indicating presence of cestodes in subcutaneous space. (b) Internal lesions associated with the plerocercoid scolex revealed upon removal of the skin.

Since the first observations of cestode infections in 2010, field protocols for snake captures have included a visual screening for external signs of parasite infection (i.e. presence or absence of blister-like raised nodules under the skin; e.g., Fig 1a). During the course of a study investigating snake predation on the endangered Mariana Swiftlet (*Aerodramus bartschi*) within the Naval Ordnance Annex, Naval Base Guam, we collected a large sample of snakes from within the known region of cestode occurrence examined these individuals for external and internal signs of infection. We also compared field-screened data with in-lab necropsy data to determine rates of false positives and negatives in field-collected data. Necropsy observations revealed that 14 of 222 *B*. *irregularis* infections detected in the lab went undetected in the field (i.e. a 6% false negative rate), and there were no cases where field-detected infections were not verified by necropsy. Therefore we used necropsy records for all analyses. Because our survey efforts for parasites were curtailed by the Swiftlet study, we recorded only parasite presence or absence for each snake for this initial study.

We collected snakes via trap or hand capture using headlamps or night vision goggles [19–20]. For each necropsied snake we measured snout-vent length (SVL) and weight prior to euthanization by blunt force head trauma immediately followed by decapitation. Snake collection and euthanization techniques were approved by the Institutional Animal Care and Use Committee (IACUC) of the U.S. Geological Survey Fort Collins Science Center (Protocol 2014–02) in accordance with humane practices for reptiles as outlined by the American Veterinary Medical Association (AVMA). We identified infected snakes based on the presence of blister-like nodules beneath the skin, which signaled the presence of cestodes (Fig 1a). We then removed the skin off the entire body and examined the musculature (Fig 1b), body cavity, and viscera for parasites.

We hypothesized that infected snakes might suffer reduced reproductive output. Although any one of several traits could potentially signal this effect (e.g. relative clutch size, clutch mass, ovarian follicle size), we measured testis volume in males and mean diameter of previtellogenic or vitellogenic ovarian follicles (> 5mm) in females and compared these traits in infected vs. non-infected snakes. We corrected for body size in our measure of testis volume by using residuals from a regression of log(testis volume) by log (SVL). We did not correct follicle diameter for snake length, as follicular development is a function of reproductive status rather than body size. We hypothesized that parasitized individuals would have smaller testis volume and smaller mean follicle diameter on average compared to non-parasitized individuals, perhaps due to hormonal suppression (e.g. testosterone and estrogen) as a response to acute stress [21]. Although limited data show no effect of some forms of parasitism on the reproductive output of female snakes [14], at least one malarial form of parasitism has been shown to decrease testis volume in male lizards [21]. Given that variation in testis volume has been linked to fitness in some taxa (i.e. males with large testes have higher reproductive success than those with small testes [22–23]), our comparative approach and the choice of traits examined here represents a reasonable starting point for investigating potential reproductive effects of infection in the invasive *B*. *irregularis* population.

### Specimen Collection, Sequencing, and Phylogenetic Analysis

We collected several individual larvae from snakes within an hour of euthanasia and extracted specimens’ muscle tissue by gently pulling along the strobila until the embedded head of the worm released. We preserved worms individually in 95% ethanol or 10% formalin for later morphological identification and DNA sequencing.

All specimens collected for this study were plerocercoid larvae and lacked a true scolex. This complicates species identification based on morphology because virtually all distinguishing taxonomic characters are drawn from the scolex. Therefore, we compared DNA sequence data from the mitochondrial cytochrome c oxidase subunit 1 gene (*cox* 1) in our samples to published *cox* 1 sequences from other Pseudophyllid cestodes. We sequenced *cox* 1 because this has been the most widely used marker in previous phylogenetic studies on these worms.

We extracted genomic DNA from whole larval specimens using a Qiagen DNeasy Blood & Tissue Kit and amplified a portion of *cox* 1 using the universal primers JB3 and JB4.5 [24–26]. We followed Liu *et al*. [25] for PCR reagent concentrations, volumes and cycling conditions. DNA sequencing was performed using Sanger methods and Big Dye v3.1 chemistry on an ABI3730XL DNA analyzer at Genewiz (La Jolla, CA). We edited the data using Sequencher v5.1 (Gene Codes Corporation, Ann Arbor, MI) and manually aligned sequences by eye. The *cox* 1 sequences are available on GenBank (see S1 Appendix for accession numbers).

To examine the placement of our samples within the broader phylogenetic context of Pseudophyllid cestodes, we aligned our data with 68 published sequences from GenBank and reduced the dataset to unique haplotypes only for phylogenetic analysis (see S1 Appendix for a summary of the haplotypes). In addition to *Spirometra*, we included sequences from species in the following genera as outgroups: *Sparganum*, *Diphyllobothrium*, *Ligula*, *Echinococcus* and *Taenia*. We selected the best-fit nucleotide substitution models and different codon partitioning schemes using Bayes Information Criterion (BIC) scores in PartitionFinder v1.1.0 [27] and performed phylogenetic analyses using BEAST v2.1.3 [28]. Phylogenetic analyses consisted of two separate runs of 3 X 10^7^ generations each (10% of the initial samples discarded as burn-in). We used an uncorrelated relaxed clock model [29–30] and a Yule Process tree prior. After verifying that model parameters had converged to similar values between runs and that effective sample sizes exceeded 200, we combined data from the posterior distributions of each run to generate a maximum clade credibility tree. Convergence statistics were evaluated in Tracer [31].

### Prevalence and Individual Probability of Infection

We report prevalence as the proportion of infected individuals from a sample. We use ‘probability of infection’ in a statistical sense, i.e., the probability that a sampled individual had a positive infection status, as opposed to predicting the probability of an uninfected snake becoming infected. An individual snake’s probability of being infected may be influenced by factors intrinsic or extrinsic to the snake. We assessed probability of infection using logistic regression; potential covariates and hypotheses for their effects include:

1. Snake body size: Since growth is indeterminate in snakes, body size is often a proxy for age. These cestode infections are chronic in nature—once an individual is infected it tends to remain infected indefinitely. Therefore, older or larger snakes are more likely to have been exposed and infected. We measured snake snout-vent length (covariate: SVL) by gently stretching snakes along a tape measure.
2. Sex: We tested for any potential sex-bias in infection rates. Sex determination was made by visual examination of the internal reproductive anatomy upon necropsy.
3. Residual body mass: Deviations from a model predicting the relationship between body length and body mass are commonly considered to be an index of an individual’s body condition [32–33]. Snakes that are heavy relative to their length are considered to be in better nutritional status with a more robust somatic mass (e.g. water content, protein, and muscle mass) associated with a successful long-term feeding history, while lighter snakes are considered to be in poorer condition. We hypothesized that infected individuals may be in poorer body condition as a relative fitness cost associated with infection. To test this hypothesis, we first calculated mass as the entire snake mass adjusted by subtraction of any stomach content and intraperitoneal fat masses. We then measured each individual’s residual body mass (RBM) as the standardized residuals from a fourth-order polynomial regression of log (mass) by log (SVL).
4. Fat mass ratio: In addition to RBM, mass of intraperitoneal fat masses relative to body mass may be an indicator of access to short-term energy stores resulting from more recent feeding success, which can translate to increased growth rates, activity, reproductive success, and survivorship [34–36]. Fat mass ratio (FMR) was calculated as the ratio of the log of the mass of fat bodies recovered from the coelomic cavity during necropsy (+1 to avoid taking the log of zero values) to the log of the adjusted mass (minus stomach contents and fat bodies). We hypothesized a negative relationship between FMR and infection, suggesting poorer short-term energy stores for infected snakes as a fitness cost associated with infection.
5. Site: While all snakes undergoing rigorous screening were sampled from within the Naval Ordnance Annex, they were collected at nine different sampling foci. As minor ecological heterogeneity exists among these sites, we hypothesized that this could lead to site-by-site variability in infection prevalence. We included a categorical covariate for site (SIT) to investigate whether sampling location had an explanatory relationship with prevalence.
6. Season: Guam’s annual climatic regime is characterized by a rainy wet season (June through November) and a warmer dry season (December through May). Given the role of standing water in the typical life cycle of spirometrid cestodes, we considered a possible effect of season (SEA) on infection prevalence.

Based on these candidate covariates we considered our global (most highly-specified) logistic regression model to be:

$$logit\left(\hat{p}\right)=\beta_{0}+\beta_{SVL}+\beta_{SEX}+\beta_{RBM}+\beta_{FMR}+\beta_{SIT}+\beta_{SEA}$$

where $\hat{p}$ is the predicted probability of infection for an individual snake. To achieve the benefits of a balanced model set [37–38], including comparisons of relative variable importance (RVI) values, all possible combinations of model terms were compared using Akaike’s Information Criteria corrected for small sample size (AICc). RVI is calculated as the sum of the AICc model weights for all models including the respective term; therefore, a term that was included in models carrying 100% of AIC model weights would have an RVI of 1.0. A top model was then selected based upon model comparisons and relative significance of component terms.

We also hypothesized that infection status may influence reproductive condition as a potential fitness cost of parasitism. Using the top prevalence model, we created sex-specific data subsets and tested relationships between infection status and residual testes volume (RTV) for males and presence of enlarged follicles (FOL) for females.

## Results

### Parasite Identification

Nodules under the skin were composed of one to several individual worms. Although we did not quantify individual infection intensity throughout the screening process, we removed as many as 157 individual cestodes from one *B*. *irregularis* with a particularly high number of skin nodules. Scanning electron micrographs of the gross morphology of specimens (see S1 Fig in Supporting Information) confirmed that the life history stage of the worm infecting *B*. *irregularis* is a plerocercoid larva in the genus *Spirometra*.

Sequencing five larvae from three different *B*. *irregularis* on Guam revealed a single COX1 haplotype (417 base pairs), and nucleotide BLAST searches on GenBank showed a 100% identity match between this haplotype and six isolates of *S*. *erinaceieuropaei* (>94% query coverage) from southern China. A single haplotype recovered in Thailand also had a 100% match to the Guam haplotype, but the query coverage was substantially lower (83%) due to the shorter length of the Thai sequence. The remaining closest matches (≥94% identity) were all isolates of *S*. *erinaceieuropaei*, thus we had high confidence that species identity of the larvae was *S*. *erinaceieuropaei*.

For phylogenetic analysis, the best-fit model-partitioning scheme favored separate substitution models for each codon position (see S2 Appendix). Assigned models and summary statistics are provided in the Supporting Information. Our tree shows strong support for the monophyly of all haplotypes attributed to *S*. *erinaceieuropaei*, and that members of the genus *Sparganum* are sister to *Spirometra* (but with low posterior probability; see S2 Fig). We recovered two well-supported clades within *S*. *erinaceieuropaei*, but neither showed any clear patterns based on geography. The Guam haplotype was nested among several others from the Guangdong Province of southern China, but the limited number of substitutions distinguishing the different haplotypes did little to resolve the relationships among them (S2 Fig). Nonetheless, it is clear that the source of *Spirometra* on Guam is in Asia, most likely southern China.

### Range and Prevalence of Infection in Brown Treesnakes

**Fig 2:**
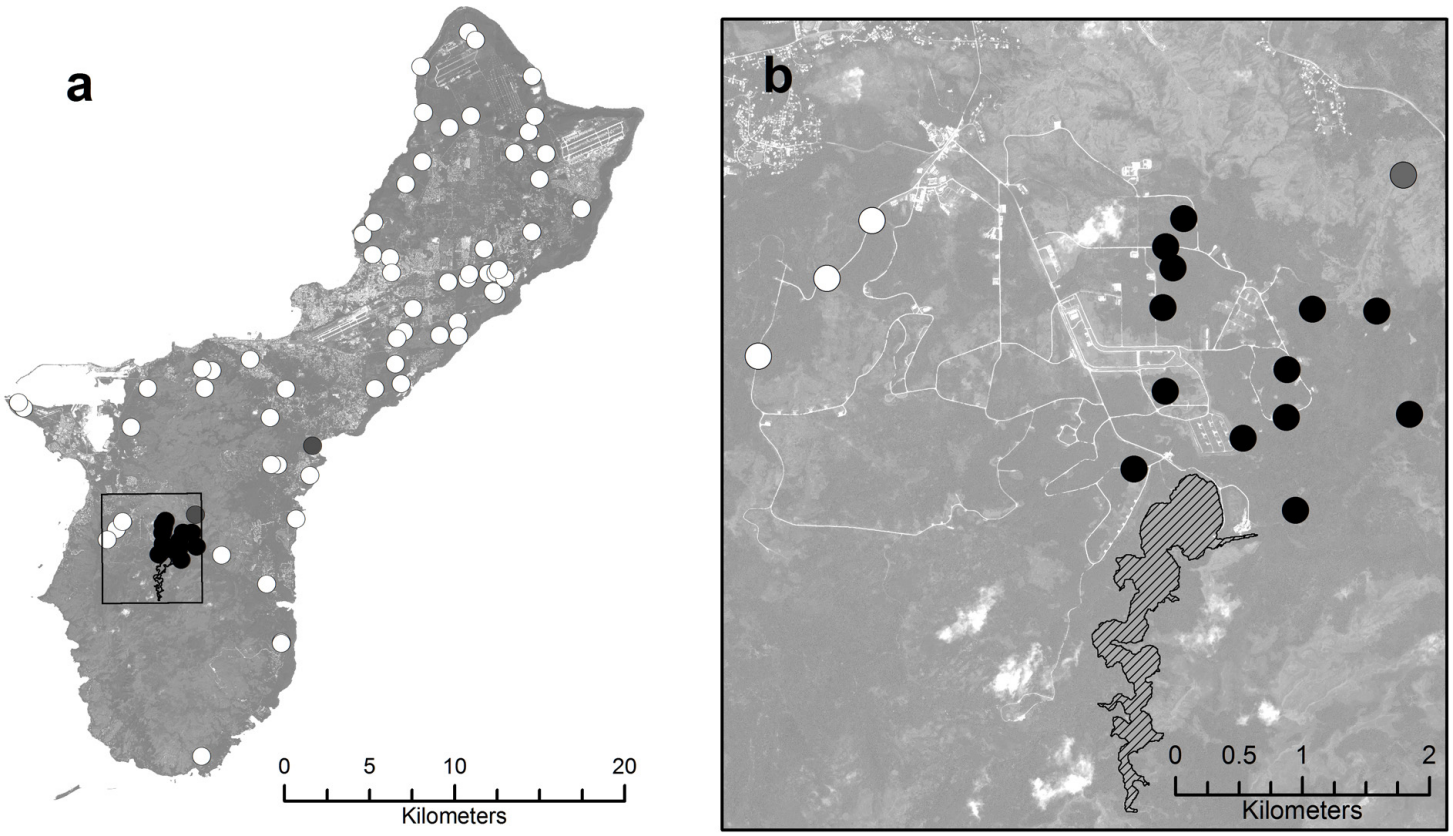
*Spirometra erinaceieuropaei* Infection Prevalence Map of Guam. (a) Locations of 77 samples of *B*. *irregularis* screened for parasite infection (N = 4 to 103 per site). White symbols denote locations where no infected snakes were found and black symbols represent locations with high infection rates. The two gray symbols represent samples of 100 snakes that contained a single infected specimen each. (b) Inset map depicting distribution of sampling sites within the area of high occurrence (Naval Ordnance Annex). Non-infected samples depicted by white spots are on an elevated limestone forest plateau, while the highly infected sites depicted by black symbols are in low-lying ravine forests. The low-infection gray symbol denotes a sample collected in savanna grassland habitat. The hatched polygon represents Fena Reservoir, the only large body of fresh water on Guam.

Screening of more than 2,700 snakes from 77 sites throughout the island between 2010 and 2014 failed to document the occurrence of this parasite in any region other than the Naval Ordnance Annex, with the exception of two sites where a single infected snake was found in each sample of 100 snakes (prevalence = 1%). These exceptions were the Pago River ravine forest site in east-central Guam and a savanna grassland site neighboring the Naval Ordnance Annex (Fig 2). Among 222 rigorously-screened *B*. *irregularis* from nine sites within the Ordnance Annex ($\bar{X}$ = 24.6 snakes per site, SD = 13.6, range = 1 to 44), the overall prevalence of infection was 77.5%. Among the seven sites providing samples of 24 snakes or greater, prevalence ranged from 54.2% to 96.7%.

### Variation in the Probability of Infection

The top 12 models describing per capita probability of infection for the Naval Ordnance Annex sample carried 95% of all AIC model weights (Table 1). The top model, carrying 40% of the model weights, included only body size (SVL), residual body mass (RBM) and relative fat mass (FMR) terms. The next three models, which included coefficients for sex or season (or both), comprised an additional 40.5% of the model weights. As each of these latter terms was added to the model, the ΔAICc value increased by ~2 AICc units, the penalty for addition of a single parameter. This indicated that neither sex nor season coefficients added explanatory value to the models [39]. This conclusion is supported by the non-significant *P*-values associated with model-averaged coefficients (P_SEX_ = 0.42, P_SEA_ = 0.87).

**Table 1:** Top AICc model results for factors associated with *Spirometra* infection (models carrying 95% of model weights).

Model	K	AICc	ΔAICc	Weight
**SVL+RBM+FMR**	4	160.9	0.00	0.398
**SVL+RBM+FMR+SEX**	5	162.3	1.43	0.195
**SVL+RBM+FMR+SEA**	5	162.9	2.06	0.142
**SVL+RBM+FMR+SEX+SEA**	6	164.4	3.54	0.068
**SVL+RBM**	3	165.3	4.44	0.043
**SVL+RBM+FMR+SIT**	12	166.5	5.65	0.024
**SVL+RBM+SEX**	4	167.1	6.20	0.018
**SVL+RBM+SEA**	4	167.2	6.36	0.017
**SVL**	2	167.3	6.42	0.016
**SVL+RBM+FMR+SEX+SIT**	13	167.7	6.85	0.013
**SVL+SEX**	3	168.4	7.56	0.009
**SVL+FMR**	3	168.5	7.59	0.009

K = number of parameters; SVL = snout-vent length; RBM = residual body mass; FMR = fat mass ratio; SEX = sex; SEA = season; SIT = site.

Body size (SVL) had the most explanatory value, with a RVI of 1.0 (included in all top models) and a model-averaged P-value of << 0.001. As predicted, larger snakes were more likely to be infected than smaller snakes. Residual body mass (RBM) was significantly and positively correlated with prevalence, being included in models carrying 92% of AICc weights and having a P-value of 0.012. The final term with significant explanatory power was the fat mass ratio (FMR), with an RVI of 0.88 and a P-value of 0.022. As predicted, prevalence was negatively correlated with relative mass of internal fat bodies. Post hoc comparisons of prediction intervals for all sites with reasonable sample sizes (over 20) confirmed no meaningful differences in prevalence among sites within this small high-prevalence region.

Based upon these results, the most parsimonious model describing factors significantly linked to infection prevalence is:

$$logit\left(\hat{p}\right)≅\;\beta_{0}+\beta_{SVL}+\beta_{RBM}+\beta_{FMR}$$

**Fig 3:**
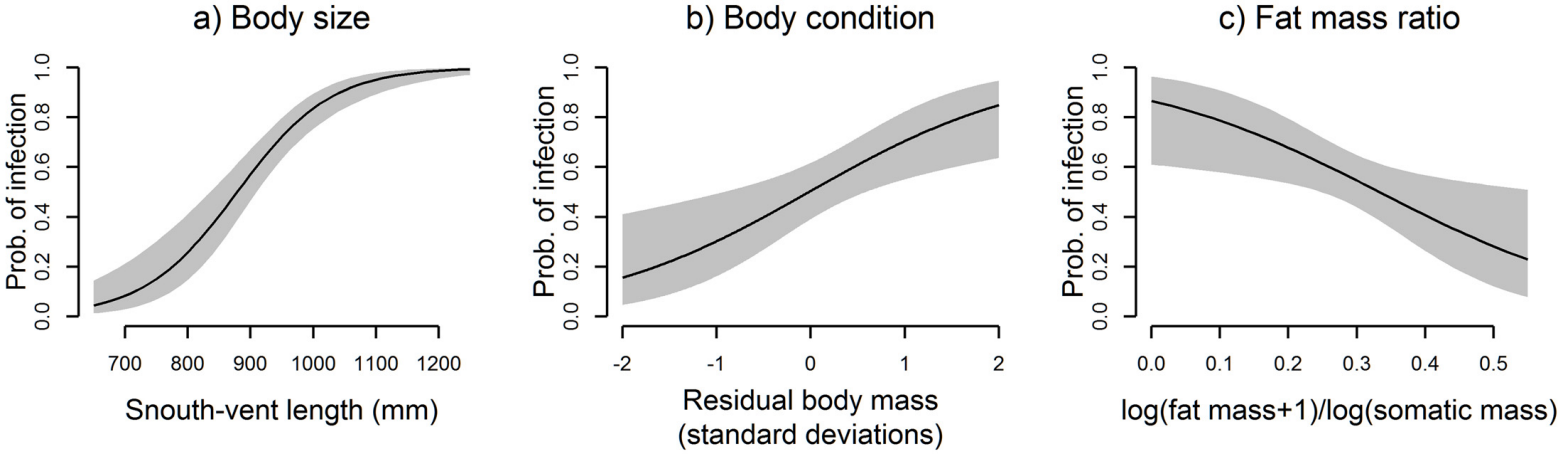
Effect sizes for model terms under the top model. (a) Effect of SVL at mean values of body condition and fat mass ratio; probability of infection = 0.5 at 880 mm SVL. (b) Effect of residual body mass at 880 mm SVL and mean fat mass ratio. (c) Effect of fat mass ratio at 880 mm SVL and mean residual body mass.

Under this model, all terms have significant P-values (P_SVL_ << 0.001, P_RBM_ = 0.004, P_FRM_ = 0.017). Effect sizes of predictor variables are depicted in Fig 3. Additional terms describing reproductive development to models based on sex-specific data subsets (residual testes volume, presence of enlarged follicles) revealed no relationship with infection status (P_RTV_ = 0.359; P_FOL_ = 0.991).

## Discussion

Our findings offer a possible example of how purely ecological processes involving serial introductions of multiple invasive host species, rather than the more typical shared co-evolutionary or historical biogeographic processes between hosts and their parasites, may be shaping new host-parasite dynamics on the Pacific Island of Guam. While it is possible that the interaction between *Spirometra* and *B*. *irregularis* represent an ‘evolutionary reunion’ rather than a novel interaction, the amount of time since the snakes’ introduction on Guam and the genetic evidence showing that the source populations of both the host and parasite are from different geographic areas and that *B*. *irregularis* has experienced a severe bottleneck (as a consequence of a founder event), suggests that co-evolutionary ties between the two have been disrupted. Furthermore, although the effects of *Spirometra* on the fitness of introduced *B*. *irregularis* have yet to be thoroughly explored, our findings suggest that a natural population control mechanism (i.e., parasite challenge) may be in progress for at least one small portion of the island that supports freshwater habitat. The circumstances leading to this control mechanism may involve a fortuitous set of additional, non-native host introductions, with the founder(s) of at least one of these host species likely harboring *Spirometra*. It is possible that infection in *B*. *irregularis* represents a spill-over and is not a necessary part of the parasite’s life cycle, but completion of ongoing studies are needed to confirm whether this is the case.

The gross morphology of plerocercoid larvae recovered from snakes was consistent with cestodes in the genus *Spirometra*. Spirometrid cestodes have a worldwide distribution, with most human infections (manifesting as the condition “sparganosis”) recorded in Southeast Asian countries [40]. They are also known to infect wild snake species across Asia, but have not been formally documented in *B*. *irregularis*. Most investigations of *Spirometra* prevalence in snakes have focused on food markets in China—where wild-caught snakes are a popular traditional food—to assess risk of sparganosis [41–42]. Little is known about the ecological and physiological effects of *Spirometra* infection in wild snakes, particularly as intermediate hosts, with most studies focusing on mammalian intermediate hosts [43–45].

While we expected *Spirometra* infection to adversely affect body condition in invasive *B*. *irregularis*, evidence from other *Spirometra* species in rodent intermediate hosts indicate that infection may have the potential to stimulate body growth via the release of a mammalian hormone-like substance called plerocercoid growth factor (PGF) [43–45]. Additional studies are needed to test whether this cesode impedes, increases, or has no effect on growth in a snake host. We found mixed evidence that infected snakes suffered fitness consequences, at least based on the limited set of traits we measured here. The only potential fitness measure that was negatively influenced by infection was the fat mass ratio, which showed that infected snakes, on average, had smaller intra-peritoneal fat reserves. This observation could be the result of the parasite diverting nutrients that might otherwise contribute to the host’s fat reserves or may cause lipodystrophy in the host, although further studies are needed to determine whether that is the case and if so, the mechanism by which it might do this.

In contrast to fat mass, we found that body condition, as represented by residual body mass, was positively associated with infection, the opposite of what we predicted for snakes under physiological duress but consistent with studies of intermediate mammalian hosts. However, for two snakes of the same length, the more robust one has likely had more lifetime feeding success, therefore exposing it to more opportunity for infection. Similarly, as we had hypothesized, longer and presumably older snakes had more lifetime feeding opportunity than shorter snakes, resulting in the predicted positive correlation with infection. These findings are consistent with some studies that have shown that high parasite infection intensities appear to have negligible fitness cost to the host [46].

In this study we focused on the association of *S*. *erinaceieuropaei* infection and fitness traits of *B*. *irregularis*. However, it is possible that physiological or behavioral traits of snakes affect their susceptibility to infection. Snakes exhibiting high individual foraging success may have more opportunity for infection via infected intermediate hosts, and snakes of differing body conditions may occupy different niches within a heterogeneous habitat (e.g. larger snakes may forage on the ground more than smaller snakes [47]). Behavioral modification has also been documented in several helminth species, which is presumably advantageous for trophic transmission when behavioral modification by a parasite increases the likelihood a host will be preyed on [48–49]. Behavioral modification in intermediate hosts infected with *Spirometra* has not been documented among herpetofauna but some species of *Spirometra* may activate growth hormones in infected mammalian hosts, potentially affecting behavioral characteristics through indirect effects on host phenotypes [43–45]. Mortality associated with infection could also influence the demography of infected snakes but we have little reason to believe *S*. *erinaceieuropaei* is causing mortality in *B*. *irregularis* on Guam, given that we have not observed any dead snakes during trap or search efforts and invasive *B*. *irregularis* have no natural predators on the island.

As we only recorded parasite presence and not parasite intensity, there may be fitness costs associated with high parasite intensity that were not reflected in our analysis. For example, we found no relationship between infection status and residual testes volume or the presence of enlarged follicles; however, many other aspects of reproductive success could be influenced by the degree of *Spirometra* infection, such as mating vigor, courtship, mate selection, or offspring life-history traits [50–54]. The known effects of lipodystrophy on activity and reproductive success on both sexes suggests that infection may come at a reproductive cost in this invasive snake population, particularly if the parasite is recently introduced to Guam, and suggests merit for further investigation in this system [55].

There is keen interest in applying new management tools to control the invasive snake population on Guam. Investigations into potential pathogens for biological management have been under recent consideration, given that numerous aspects of the snake’s population biology on Guam and other attributes of the island itself (e.g. size, location and absence of native snake species) are favorable for this form of management [9,18,56–58]. Nevertheless, use of this parasite for island-wide biological management would most likely not be effective given that freshwater is necessary to complete the *Spirometra* life cycle. To date, *Spirometra* infections are restricted to the low-lying, moist ravine forests within the Naval Ordnance Annex region due to a unique topography and hydrology that supports freshwater year-round. Natural spread of this parasite across the island is unlikely because most of the habitat on Guam consists of elevated limestone plateaus devoid of perennial freshwater.

**Fig 4:**
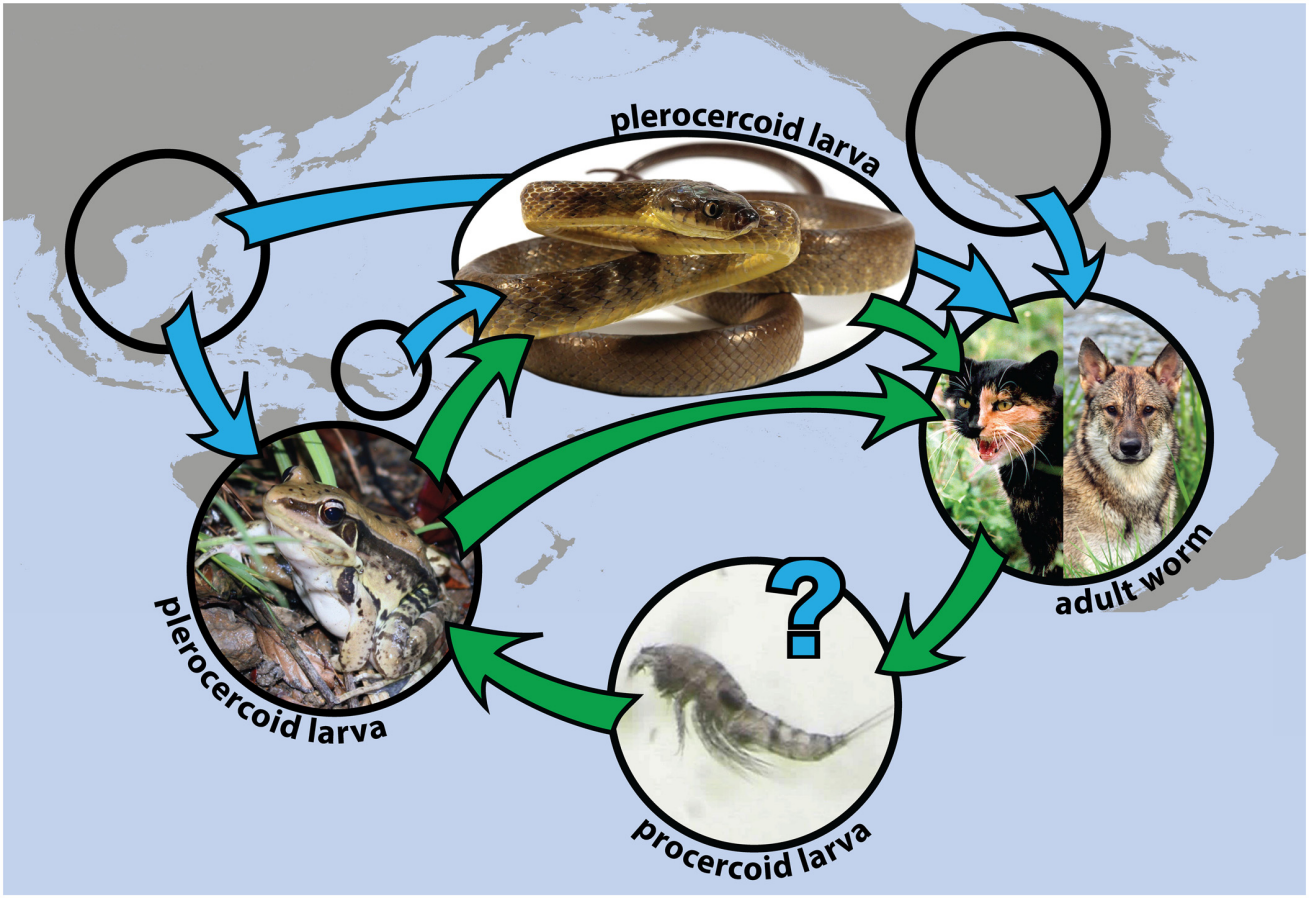
Proposed Life Cycle Diagram of *Spirometra erinaceieuropaei* on Guam. Blue arrows show hypothesized hosts’ geographic source population, green arrows indicate *S*. *erinaceieuropaei* life cycle stages as represented by hosts, and life history stages are labeled under each host. Eggs hatch in fresh water where they are ingested by copepods, which are in turn ingested by aquatic vertebrates such as frogs and fish. *Spirometra* mature into adult worms when a second intermediate host is consumed by a carnivorous mammal (typically feral cats (*Felis catus*) and dogs (*Canis lupus familiaris*), both of which occur on the Naval Ordnance Annex), and the life cycle is completed when mammals shed feces containing ova into fresh water. Photo credits listed in Acknowledgments.

Future studies are needed to determine the full suite of hosts sustaining the life cycle of *S*. *erinaceieuropaei* on Guam, and whether these hosts are native or introduced. However, based on the generalist life cycle of this genus, along with their isolated distribution on Guam, we can make hypotheses about which species may support the parasite (Fig 4). *Boiga irregularis* likely ingest infected frogs that contain the developing plerocercoids, as frogs are a typical intermediate host for *Spirometra*. The life cycle is complete when the definitive host (*F*. *catus* or *C*. *lupus familiaris*) eats an infected intermediate host [42, 57]. We have observed frog remains (>3 individual frog skulls) in the stomach of a vehicle-killed feral cat that was collected on the Naval Ordnance Annex. No adult *S*. *erinaceieuropaei* were found in the intestine of this individual, but it is quite possible that cats are ingesting infected frogs or snakes.

The larval stage, host pathology, and complex life cycle of *S*. *erinaceieuropaei* found in *B*. *irregularis* suggest that these snakes are second intermediate hosts [40,57]. Field observations, including a plerocercoid parasitic cyst found in the hind limb muscle tissue of an invasive frog species, *Fejervarya cancrivora*, collected from the Naval Ordnance Annex indicate that frogs also host the flatworm (see S3 Fig). *Spirometra* have also been observed in frogs in parts of Asia [25,56]. Both *F*. *cancrivora* and another invasive frog species, *Hylarana* (*Sylvirana*) *guentheri*, are abundant in the area where *S*. *erinaceieuropaei* occurs on Guam. Predation on these frogs by *B*. *irregularis* has been observed through stomach necropsy, and frogs may be the pathway by which snakes become infected.

Both *H*. *guentheri* and *F*. *cancrivora* have been introduced to Guam, presumably via the aquaculture trade (fish, crustacean, and mollusc shipments for human consumption) from China and the Philippines in 2001 and 2005, respectively [11]. *Spirometra* has a global distribution and is known to occur in the highest frequency in these regions of Asia [40,42]; however for this system, further work in the native range of *S*. *erinaceieuropaei* is needed to better pinpoint the geographic origin of the introduction, a task that could be aided by simultaneously identifying a source for the introduced frog species. Although it is unknown whether *B*. *irregularis* translocated this parasite to Guam during the founder event(s), it seems highly unlikely given that *S*. *erinaceieuropaei* was only detected in 2010, despite several decades of intensive study on this population and the conspicuous signs of even minor infection in snakes.

Our study supports the hypothesis that novel assemblages of exotic species can result in a shift from parasite-host interactions driven by long-term evolutionary processes, to interactions driven by ecological processes developing on short temporal scales [4]. In no instance has a native species been implicated in the life cycle of this recently discovered parasite on Guam, which has no native amphibians or carnivorous mammals. The least-known link in the spirometrid life cycle on Guam is the copepod intermediate host, which may or may not be an introduced species. Since the aquacultural trade has been implicated in the introduction of frogs to Guam [11] it seems possible that the copepods accompanied the frogs in shipments of the same materials. Therefore, we consider it entirely plausible that the establishment and life cycle of this recently discovered parasite was enabled by multiple introductions of different non-native species to this remote Pacific island.

## Supporting Information

S1 Appendix Summary of *cox*1 haplotypes. Numbers in brackets following the different haplotype labels are GenBank accession numbers.
(DOCX)

S2 Appendix Results of analyses for substitution model selection and model partitioning schemes for phylogenetic reconstruction. Analyses were performed using PartitionFinder v1.1.0.
(DOCX)

S1 Dataset Logistic regression prevalence dataset. Original data used in infection prevalence analyses.
(XLSX)

S1 Fig Scanning electron microscope (SEM) images of plerocercoid larvae of *Spirometra erinaceieuropaei* collected from three differenct *Boiga irregularis* on Guam. (a) Inverted distal tip of the strobili, which lacks the true scolex of adult worms. (b) Close-up image showing the inverted distal tip. (c) Close-up image of the plerocercoid strobila shown in (a)-(b). (d) Strobila of a second plerocercoid where beginnings of segmentation are apparent. (e) Strobili of a third plerocercoid individual. (f) Close-up showing specialized microvilli (i.e. microtriches) covering the entire surface of the tegument of the worm and used for nutrient uptake. Scale bars: (a) 500 μm; (b) 50 μm; (c)-(d) 200 μm; (f) 5 μm.
(TIF)

S2 Fig Maximum clade credibility tree for *cox*1 haplotypes of *Spirometra* and outgroup species. Bullets indicate well-supported branches with posterior probabilities ≥0.90. Numbers in parentheses following the outgroup names are Genbank accession numbers for the *cox* 1 haplotypes.
(TIF)

S3 Fig Plerocercoid parasitic cyst found in the hind limb muscle tissue of an invasive frog species, *Fejervarya cancrivora*, collected from the Naval Ordnance Annex. (TIF)

S1 Metadata Meta data for logistic regression prevalence dataset. Metadata file for S1 Dataset.
(TXT)
