# Supplementary material for: Invaded Invaders: Infection of Invasive Brown Treesnakes on Guam by an Exotic Larval Cestode with a Life Cycle Comprised of Non-Native Hosts
Source: PLoS One. 2015 Dec 23;10(12):e0143718. doi: 10.1371/journal.pone.0143718 (PMC4689450; doi:10.1371/journal.pone.0143718)
Supplement: S1 Appendix — Numbers in brackets following the different haplotype labels are GenBank accession numbers. (DOCX) [file pone.0143718.s001.docx]

**S1 Appendix. Summary of *cox*1 haplotypes.** Numbers in brackets following the different haplotype labels are GenBank accession numbers.

Number of sequences: 68

Number of sites: 417

Number of haplotypes, h: 24

Haplotype diversity, Hd: 0.8630

*Spirometra* Clade 1

Hap_9: 1 [JQ267473]

Hap_10: 23 [AB374543 AB369249 GQ866868 AJ308264 AJ308265 AB278573 KF539833 GQ999951 GQ999956 AF096238 AF096237 FJ886763 FJ886764 FJ886768 KF656740 KF656739 KF656742 KF656741 AB522603 KF656736 KF656735 KF656746 KF656745]

Hap_13: 1 [AF181887]

Hap_14: 2 [KF656738 KF656737]

Hap_15: 2 [KF656744 KF656743]

*Spirometra* Clade 2

Hap_1: 6 [JQR00001(Guam) GQ866863 GQ866864 KC551943 FJ886765 FJ886769]

Hap_2: 7 [GQ999947 GQ999953 GQ866872 FJ886766 FJ886771 GQ999946 GQ999948]

Hap_3: 1 [AB369250]

Hap_4: 5 [GQ866882 GQ866884 GQ999957 HQ699076 GQ999955]

Hap_5: 5 [GQ866879 AJ308257 AJ308258 AB278577 AB480297]

Hap_6: 1 [GQ999954]

Hap_7: 2 [GQ999950 GQ999949]

Hap_8: 1 [AB27857]

Hap_11: 1 [AB369251]

Hap_12: 1 [AB015754]

Non-*Spirometra* outgroups

Hap_16: 1 [KF57295]

Hap_17: 1 [AB015753]

Hap_18: 1 [HF947568]

Hap_19: 1 [EU241247]

Hap_20: 1 [JX860633]

Hap_21: 1 [AM712906]

Hap_22: 1 [AB015755]

Hap_23: 1 [FM209182]

Hap_24: 1 [AB271234]
