# Supplementary material for: Invaded Invaders: Infection of Invasive Brown Treesnakes on Guam by an Exotic Larval Cestode with a Life Cycle Comprised of Non-Native Hosts
Source: PLoS One. 2015 Dec 23;10(12):e0143718. doi: 10.1371/journal.pone.0143718 (PMC4689450; doi:10.1371/journal.pone.0143718)
Supplement: S2 Appendix — Analyses were performed using PartitionFinder v1.1.0. (DOCX) [file pone.0143718.s002.docx]

**S2 Appendix**. **Results of analyses for substitution model selection and model partitioning schemes for phylogenetic reconstruction.** Analyses were performed using PartitionFinder v1.1.0.

Alignment: Diphyllobothrid COX1

Substitution models tested: K81+I, GTR+I, F81+I+G, GTR+G, TrN+G, F81, K81+G, F81+G, TIM, TIM+I+G, TVM+G, JC+I+G, TVMef+G, TVMef+I, TVM+I, TrNef+I, F81+I, JC, TIMef+G, HKY, K81uf+I, TVMef, K81uf+G, K81+I+G, TIMef+I, TVM, SYM+I, K81uf, JC+I, K80, K81, K80+I+G, K80+I, SYM+G, K81uf+I+G, K80+G, TrN+I, GTR+I+G, GTR, TIM+G, HKY+I+G, TIMef, TIM+I, TVMef+I+G, TVM+I+G, HKY+G, SYM+I+G, TrN, HKY+I, SYM, TIMef+I+G, JC+G, TrNef, TrNef+G, TrNef+I+G, TrN+I+G

Model selection criterion: Bayes information criterion (BIC)

Search algorithm: greedy

Best partitioning scheme

Scheme lnL: -2085.3396

Scheme BIC: 4574.89597686

Number of parameters: 67

Number of nucleotide sites: 417

Number of subsets within the data: 3 (representing each codon position)

Subset | Best Model | Subset Partitions | Subset Sites

1 | TrN+G | COX1_position 1 | 1-417\3

2 | F81+I | COX1_position 2 | 2-417\3

3 | GTR+I+G | COX1_position 3 | 3-417\3
