# Supplementary figures and images for: Invaded Invaders: Infection of Invasive Brown Treesnakes on Guam by an Exotic Larval Cestode with a Life Cycle Comprised of Non-Native Hosts
Source: PLoS One. 2015 Dec 23;10(12):e0143718. doi: 10.1371/journal.pone.0143718 (PMC4689450; doi:10.1371/journal.pone.0143718)

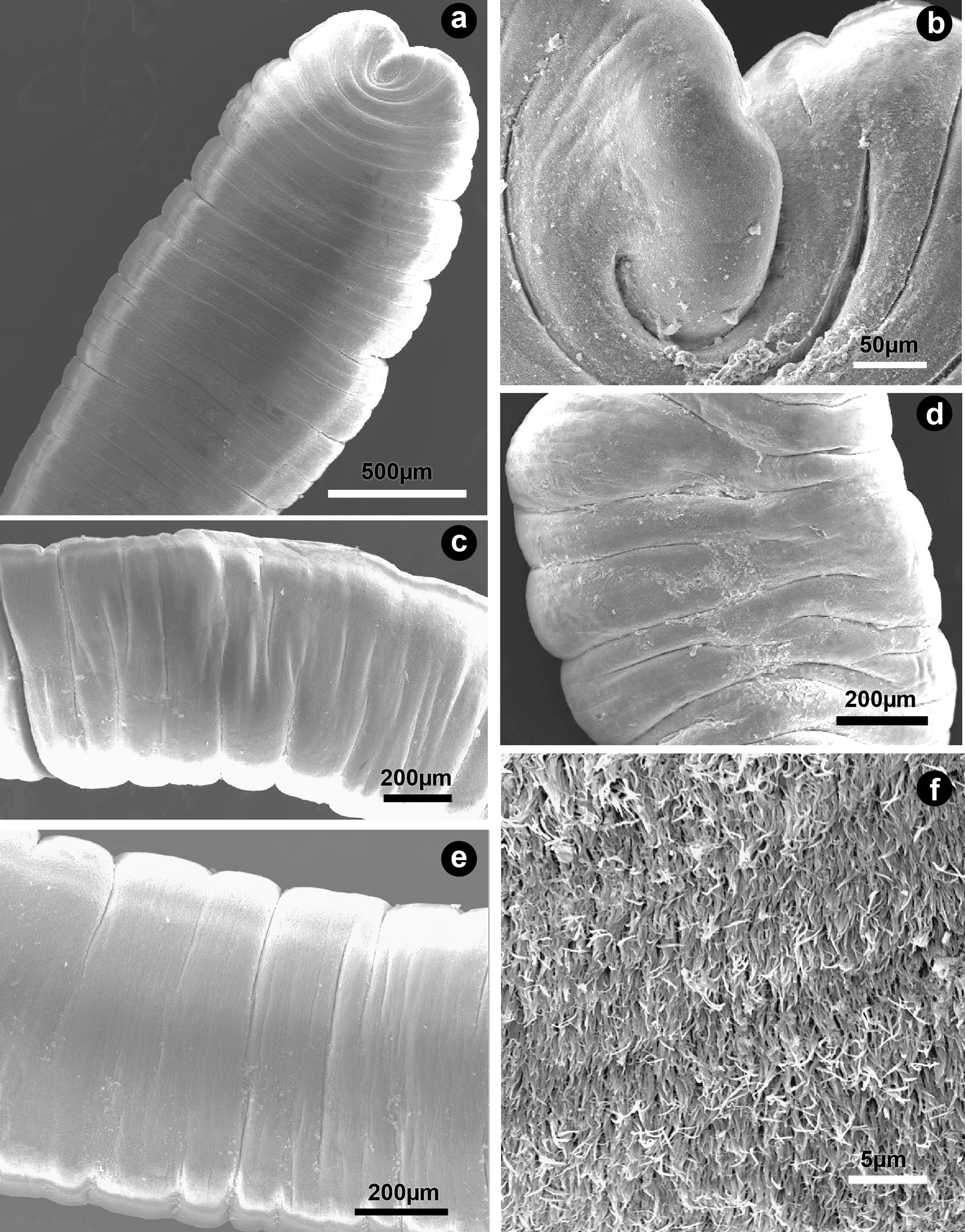

Supplement: S1 Fig — (a) Inverted distal tip of the strobili, which lacks the true scolex of adult worms. (b) Close-up image showing the inverted distal tip. (c) Close-up image of the plerocercoid strobila shown in (a)-(b). (d) Strobila of a second plerocercoid where beginnings of segmentation are apparent. (e) Strobili of a third plerocercoid individual. (f) Close-up showing specialized microvilli (i.e. microtriches) covering the entire surface of the tegument of the worm and used for nutrient uptake. Scale bars: (a) 500 μm; (b) 50 μm; (c)-(d) 200 μm; (f) 5 μm. (TIF) [file pone.0143718.s004.tif]

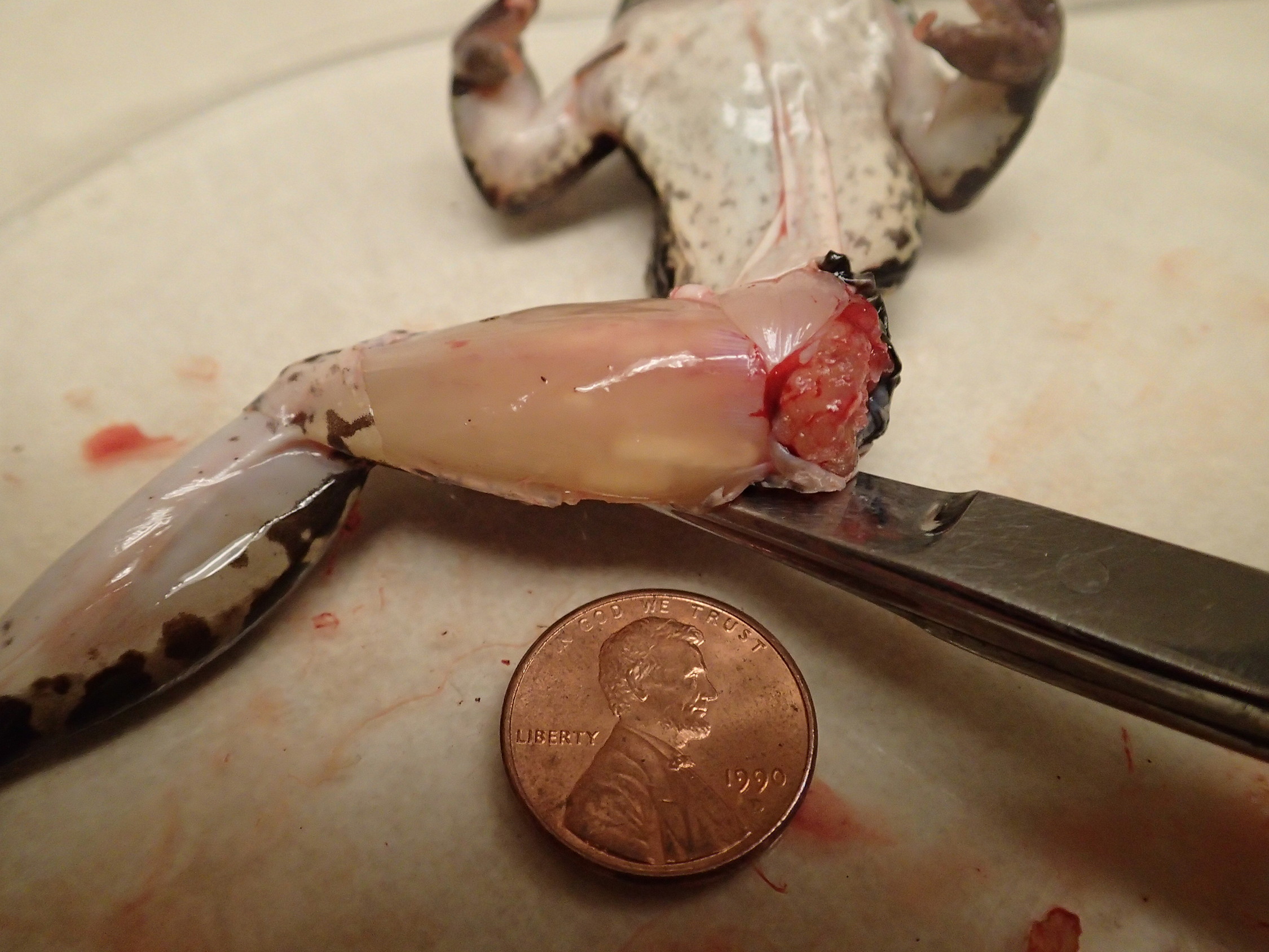

Supplement: S3 Fig — (TIF) [file pone.0143718.s006.tif]
